# Reply to Serrano et al. Comment on “Colletti et al. Prevalence and Management of Cancer of the Rectal Stump after Total Colectomy and Rectal Sparing in Patients with Familial Polyposis: Results from a Registry-Based Study. *Cancers* 2022, *14*, 298”

**DOI:** 10.3390/cancers14133241

**Published:** 2022-07-01

**Authors:** Gaia Colletti, Chiara Maura Ciniselli, Emanuele Rausa, Stefano Signoroni, Ivana Maria Francesca Cocco, Andrea Magarotto, Maria Teresa Ricci, Clorinda Brignola, Andrea Mancini, Federica Cavalcoli, Laura Cattaneo, Massimo Milione, Paolo Verderio, Marco Vitellaro

**Affiliations:** 1 Department of Surgery, Colorectal Surgery Unit, Fondazione IRCSS Istituto Nazionale dei Tumori, 20133 Milan, Italy; gaia.colletti@gmail.com (G.C.); marco.vitellaro@istitutotumori.mi.it (M.V.); 2 General Surgery Residency Program, University of Milan, Via Festa del Perdono 7, 20122 Milan, Italy; 3 Unit of Bioinformatics and Biostatistics, Department of Applied Research and Technological Development, Fondazione IRCCS Istituto Nazionale dei Tumori, 20133 Milan, Italy; chiara.ciniselli@istitutotumori.mi.it (C.M.C.); paolo.verderio@istitutotumori.mi.it (P.V.); 4 General Surgery 1, Papa Giovanni XXIII Hospital, 24127 Bergamo, Italy; erausa@asst-pg23.it; 5 Unit of Hereditary Digestive Tract Tumours, Department of Surgery, Fondazione IRCCS Istituto Nazionale dei Tumori, 20133 Milan, Italy; mariateresa.ricci@istitutotumori.mi.it (M.T.R.); clorinda.brignola@istitutotumori.mi.it (C.B.); 6 Department of General Surgery, Whipps Cross University Hospital, London E11 1NR, UK; i.cocco@nhs.net; 7 Diagnostic and Surgical Endoscopy Unit, Fondazione IRCSS Istituto Nazionale dei Tumori, 20133 Milan, Italy; andrea.magarotto@istitutotumori.mi.it (A.M.); andrea.mancini@istitutotumori.mi.it (A.M.); federica.cavalcoli@istitutotumori.mi.it (F.C.); 8 First Pathology Division, Department of Diagnostic Pathology and Laboratory, Fondazione IRCSS Istituto Nazionale dei Tumori, 20133 Milan, Italy; laura.cattaneo@istitutotumori.mi.it (L.C.); massimo.milione@istitutotumori.mi.it (M.M.)

We carefully read the comment by Serrano et al. [1] discussing the recent published article entitled “Prevalence and Management of Cancer of the Rectal Stump after Total Colectomy and Rectal Sparing in Patients with Familial Polyposis: Results from a Registry-Based Study” [2].

We thank them for the interest they have shown regarding some aspects of the study and the FAP patients’ management following prophylactic surgery with rectal sparing. They would like to know further genotype data about patients who developed cancer in the rectal stump during follow-up. We are pleased to list the genotype information of each of the 47 patients with rectal cancer in Table 1 and the patient-level heat map with the main clinical and genetic data in Figure 1. Regarding the comparison of our results with the literature, neither Colletti et al. [2] nor this author’s reply aim to act as a systematic review of the literature; thus, we do not exclude the possibility that some studies on the same topic may differ from our data. However, we still believe this to be in keeping with the current literature. Serrano et al. argued that the median interval of diagnosis of rectal cancer from primary surgery (i.e., 13 years) was consistently low compared with those in the literature. Particularly, they cited studies by Bulow [3] and Koskenvuo [4] which showed a median interval of 11 and 14 years, respectively. Despite the absence of the datasets of the aforementioned studies, our results seem to be in line with them. Moreover, Serrano et al. deem that 6.57% of patients developing rectal cancer following IRA is a very good result compared to the literature. Our results are fully in line with those reported in three studies [3,5,6], while they are quite a bit lower compared to Koskenvuo et al. [4]. 

Reading the comment, we had the feeling that Serrano et al. strongly seek a relation between genotype variant and surveillance following IRA. Thanks to a number of authors who investigated the relation between genotype and phenotype, it has been well established that number of polyps, age of onset of symptoms, colonic cancer, or extracolonic manifestations correlate with some APC mutations [7]. In fact, the aim of those studies was to categorize a subgroup of FAP patients according to genotype variant in the attempt to design better management. However, at the moment, the only significant and independent risk factor for rectal cancer following IRA is chronological age. Years after colectomy, sex, proband/call-up status, familial/isolated case, colon cancer at IRA, or location of mutation did not show enough statistical significance [3]. Based on these data, the patients undergoing IRA at the National Cancer Institute of Milan are scheduled for an endoscopic surveillance every 6–12 months, as we mentioned in the article [2]. Lastly, Serrano et al. questioned that, despite strict endoscopic surveillance, the conservative treatment was feasible only in 25pts (53%). As we stated in the article [2], strict endoscopic surveillance allows detection of rectal cancer at an early stage in the majority of patients. However, we are analyzing the data of patients who have been treated over the last 45 years in a single center, and we undoubtedly need to consider some bias. First, we need to consider that the surgical treatment has substantially shifted towards a minimally invasive approach (TAMIS) over the last two decades, and it always depends on the surgeon’s expertise and skills [8]. Moreover, in our series, some patients underwent a proctectomy because of a carpet-like rectal polyposis, although the tumor was at an early stage. However, we feel that the key perspective which should emerge is that the majority of our patients had rectal cancer detected at an early stage and were promptly treated; this scheme should dramatically improve their oncological outcomes and strengthen the IRA indication as preventive surgery [9].

## Figures and Tables

**Figure 1:**
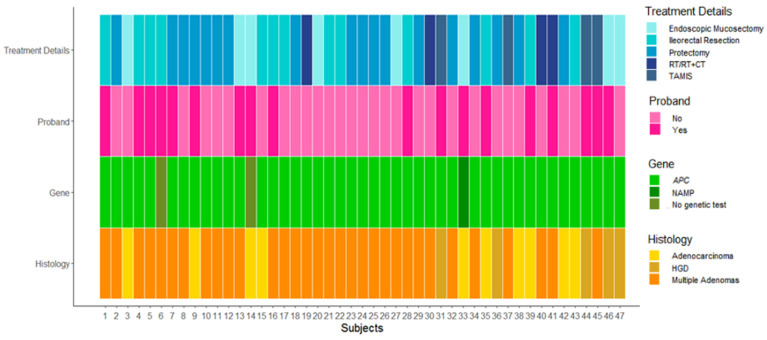
Patient-level heatmap. Representation of the genetic, baseline, and rectal stump main surgical details of the considered 47 patients.

**Table 1:** Genotype information of the 47 patients who developed a rectal cancer.

Pts Code	Protein-Coding Variants	Single Nucleotide Variants
1	p.Asp842Argfs*2	c.2523dup
2	p.Leu629*	c.18886T>A
3	p.Arg216*	c.646C>T
4	p.Arg976Lysfs*9	c.2926dup
5	p.Tyr935*	c.2805C>A
6	NO	NO
7	p.Q1294*	c.3880C>T
8	p.Gln1328*	c.3982C>T
9	p.Tyr1376Cysfs*9	c.4127_4128del
10	p.Glu1538Ilefs*5	c.4612_4613delGA
11	p.Glu1309Aspfs*4	c.3927_3931delAAAGA
12	p.Arg213*	c.637C>T
13	p.Gln1062*	c.3183_3187del
14	NO	NO
15	p.Arg640Thrfs*11	c.1917dup
16	p.Glu1309Aspfs*4	c.3927_3931del
17	p.Glu1309Aspfs*4	c.3927_3931delAAAGA
18	p.Glu1309Aspfs*4	c.3927_3931del
19	p.Thr1301Asnfs*14	c.3901dup
20	p.Glu1309Aspfs*4	c.3927_3931delAAAGA
21	p.Gln181*	c.541C>T
22	p.Glu1309Aspfs*4	c.3927_3931delAAAGA
23	p.Lys1061Asnfs*65	c.3183del
24	p.Glu1309Aspfs*4	c.3927_3931del
25	p.Glu1309Aspfs*4	c.3927_3931del
26	p.Ser1110*	c.3329C>G
27	p.Glu1309Aspfs*4	c.3927_3931delAAAGA
28	p.Ser1276*	c.3827C>G
29	p.Glu1309Aspfs*4	c.3927_3931delAAAGA
30	p.Gly471Aspfs*27	c.1409del
31	p.Arg1114*	c.3340C>T
32	p.Arg213*	c.637C>T
33	NO	NO
34	p.Asn936Lysfs*7	c.2808_2815del
35	p.Thr1556Leufs*9	c.4666delA
36	p.Glu1157Aspfs*7	c.3471_3474del
37	p.Val312Cisysfs*16	c.1312+5G>T
38	p.Lys455Glufs*5	c.1362dupG
39	p. Glu1157Aspfs*7	c.3471_3474del
40	p. Glu1157Aspfs*7	c.3471_3474del
41	p. Arg1450*	c.4348C>T
42	p.Ile544Leufs*5	c.1629delT
43	p. Glu1157Aspfs*7	c.3471_3474del
44	p.Asp1266*	c.3795_3796InsT
45	p.Lys1061Lysfs*2	c.3183_3187delACAAA
46	p.EX 11_EX 15del	Genomic reference g.(112157642_112162832)_(112179726_?)del
47	p.Gly972Valfs*4	c.2915_2916delinsTAAA
